# Detecting COVID-19-Related Fake News Using Feature Extraction

**DOI:** 10.3389/fpubh.2021.788074

**Published:** 2022-01-04

**Authors:** Suleman Khan, Saqib Hakak, N. Deepa, B. Prabadevi, Kapal Dev, Silvia Trelova

**Affiliations:** ^1^Air University, Islamabad, Pakistan; ^2^Canadian Institute for Cybersecurity, University of New Brunswick Fredericton, Fredericton, NB, Canada; ^3^School of Information Technology and Engineering, VIT University, Vellore, India; ^4^Division for Institutional Planning, Evaluation and Monitoring (DIPEM), University of Johannesburg, Johannesburg, South Africa; ^5^Department of Information Systems, Faculty of Management, Comenius University Bratislava, Bratislava, Slovakia

**Keywords:** COVID-19, fake news, social media, feature extraction, machine learning

## Abstract

Since its emergence in December 2019, there have been numerous posts and news regarding the COVID-19 pandemic in social media, traditional print, and electronic media. These sources have information from both trusted and non-trusted medical sources. Furthermore, the news from these media are spread rapidly. Spreading a piece of deceptive information may lead to anxiety, unwanted exposure to medical remedies, tricks for digital marketing, and may lead to deadly factors. Therefore, a model for detecting fake news from the news pool is essential. In this work, the dataset which is a fusion of news related to COVID-19 that has been sourced from data from several social media and news sources is used for classification. In the first step, preprocessing is performed on the dataset to remove unwanted text, then tokenization is carried out to extract the tokens from the raw text data collected from various sources. Later, feature selection is performed to avoid the computational overhead incurred in processing all the features in the dataset. The linguistic and sentiment features are extracted for further processing. Finally, several state-of-the-art machine learning algorithms are trained to classify the COVID-19-related dataset. These algorithms are then evaluated using various metrics. The results show that the random forest classifier outperforms the other classifiers with an accuracy of 88.50%.

## 1. Introduction

Reports about the novel coronavirus' (COVID-19) origin in Wuhan city in Hubei province, China came into the limelight in December 2019. Since then, the virus has spread in several provinces in China, and gradually to the majority of countries across the globe. Millions of people have been affected globally by this virus leading to the deaths of hundreds of thousands of people across the globe especially in countries like Italy, Spain, the United States, India, Brazil, and Russia as of June 2020 (1–3). This led the World Health Organization to declare COVID-19 as a pandemic in March 2020 (4, 5). It is estimated that on average, each person infected by COVID-19 infects around 2.5 persons if everyone goes on with their normal lives. Thus, the initial person infected may lead to 406 further infections per month. Hence, many nations have implemented lockdowns and social distancing to reduce the spread of COVID-19 (6).

Social media is flooded with millions of posts about COVID-19. Even though some of the information in social media is genuine and informative, most of the information spread in social media about COVID-19 is potentially rumor. Several doctored videos and photos about origin, spread of the virus, vaccines, and deaths caused by COVID-19 are being shared over social media. It is estimated that around 30–35% of the news, videos, and photos spread on social media platforms are fake. This fake news travels faster than the virus itself creating widespread panic (7).

**Figure 1:**
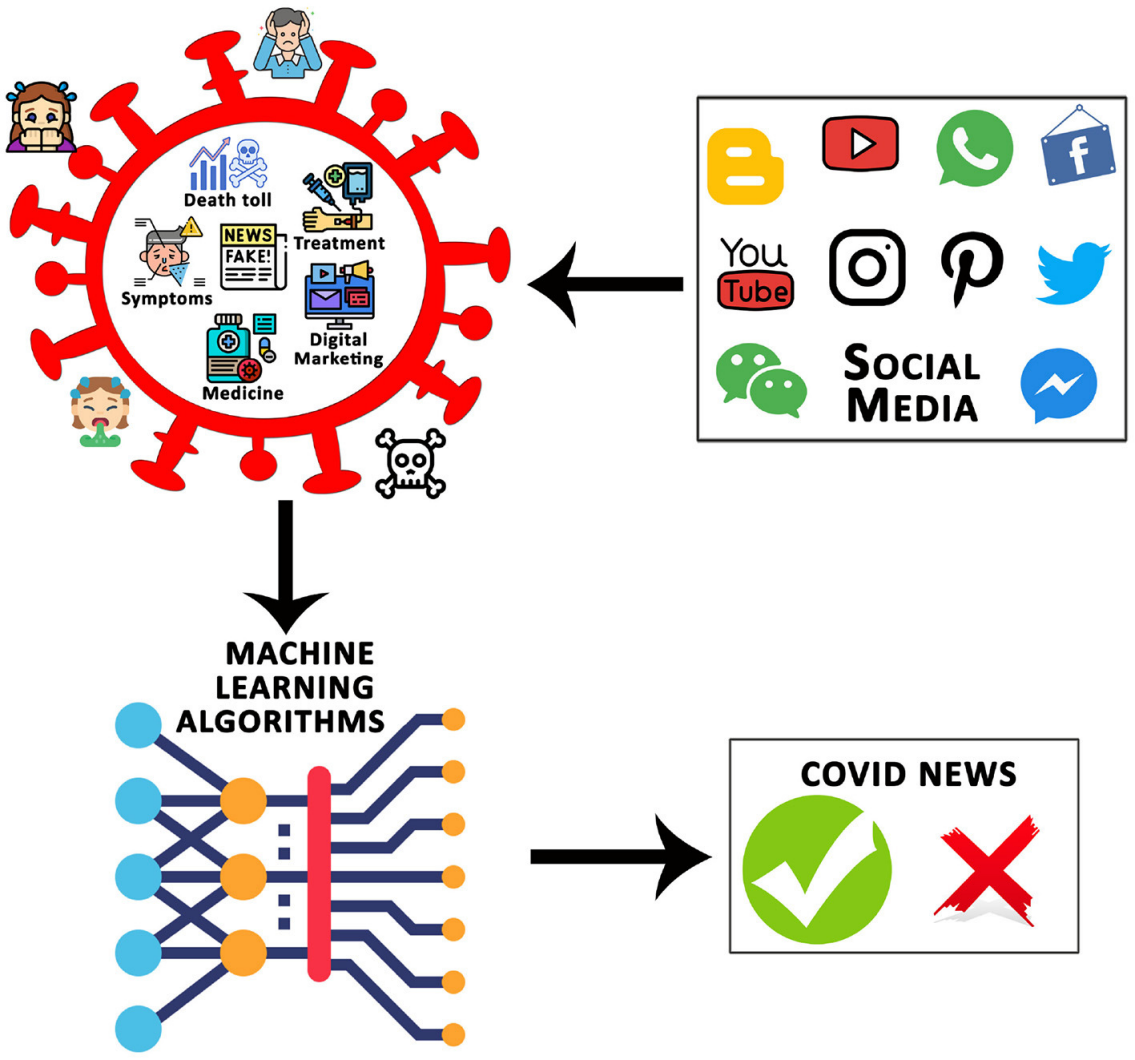
Application of machine learning algorithms for COVID fake news detection.

According to the International Fact Checking Network's (IFCN) study between January and April 2020, the fake news spread on social media can be categorized as follows: content about symptoms, causes, and cures, government documents, spread of the virus, misrepresentation of videos and photos, comments of politicians, and conspiracies that blame particular groups, countries, or communities for the spread of the virus. The fake news spread on social media has led to economic crisis in some countries. For instance, in some countries people stopped consuming non-vegetarian food as fake news was spread that animals and birds could be infected with COVID-19 and consuming non-vegetarian food may spread the virus in people. This had a severe impact on the sales of non-vegetarian food in some countries affecting the livelihood of many people (8). Figure 1 depicts the application of machine learning (ML) algorithms (9, 10) to detect fake news spread on social media.

In view of the serious impacts of the spreading of fake news which affects the privacy and security (11, 12) of users, the need of the hour is to design a successful mechanism to detect/predict fake news. In this work, an attempt is made to classify the fake news regarding COVID-19. The dataset used in this work is a fusion of fake and real news collected from several social media platforms and websites (13). This dataset is then subjected to preprocessing to remove some irrelevant text such as URL punctuations and noisy data, etc. Then, the resultant text is split into several small words using tokenization. Then, the most important features are extracted from these tokens. These features are then trained by state-of-the-art ML algorithms to classify whether the COVID-19-related news is fake or real.

The main contributions of the current work are as follows:

- Classification of a novel COVID-19 fake news dataset collected from several social media platforms.
- Generating 39 linguistic and sentiment (14–16) features from text.
- Extracting the most important features from the COVID dataset to improve the accuracy of the ML algorithms.
- An accuracy of 88.50% is achieved by the random forest classifier.

The rest of the paper is organized as follows: In section 2, related works on COVID-19 fake news are discussed. ML algorithms used in this work to classify the fake news dataset are discussed in section 3. The proposed architecture is presented in section 4. The experiment results are presented along with the discussion in section 5. Finally the paper is concluded in section 6.

## 2. Literature Survey

Artificial intelligence (AI) has made a significant contribution to different aspects of the COVID-19 outbreak. A survey on various applications of AI for fighting COVID was presented in Nguyen (17). Specifically, deep learning algorithms play a vital role in empowering AI in most of the applications with larger datasets with different types of data. Some of the applications are computer-generated for predicting infected people from a large gathering through a high-resolution image. The most prominent application is in text mining assisted with natural language processing (NLP) for extracting semantically related information from numerous texts in multilingual news data collected from various social networking platforms. The details obtained from these models such as susceptible infected recovered (SIR) models can be injected into deep learning models for the prediction of COVID-19 trends of transmission and can avoid future pandemic or epidemic diseases (18). Furthermore, a mapping study on various AI models with big data for detecting fake news on social media was carried out in Meneses Silva et al. (19). They suggested that the long short term memory model provides better results than other algorithms but incurs more processing power.

Although social media is the platform that spreads news faster with low cost than any other media among all age groups, it is progressively more dangerous in spreading fake news. Many research projects were conducted on detecting fake news from social media. A two-step model for detecting fake news from the real story in social media using artificially intelligent algorithms was designed (20). Three real datasets were considered for the study. The dataset with unstructured data was preprocessed to obtain meaningful data and was represented using vectors. In turn, the results were generated for 23 supervised learning algorithms and compared based on four evaluation metrics. On the other hand, a biostatistical analysis of the COVID-19 pandemic was carried out in Bandyopadhyay and Dutta (21) using the KNN classifier. They collected data based on certain information from the news topics in social media through multi-document summarization. This summarization method extracted the information based on the lexical information of the topic and social patterns. The KNN classifier predicted fake news with an accuracy of 80%.

Furthermore, the study in Groza (22) investigated the spread of deceptive information on social media through COVID Ontology. The reasoning in the natural language is converted into description logic for perceiving inconsistencies among different medical sources. The authors argue for an ontology-based approach for detecting fake news related to the coronavirus. The proposed model explores how well description logics (DLs) understand contradictions in figuring out whether they are true or false. The authors pay particular attention to translating the natural language into DL using the FRED translator, after which, justification is performed to test the performance of the prediction using the racer method. The proposed ontology model reveals misconceptions that circulate in digital news and shows accurate facts. However, potential research can be expanded by program evaluation and provides a verbal remedy for any conflict that exists.

Fake news or false information about this COVID-19 pandemic are very prominent and should be detected to avoid unwanted chaos. A FakeCovid dataset from a multilingual news article was generated, and a classifier was designed to detect the fake news from the dataset generated (23). BERT, the ML-based classifier model, performed better on the dataset generated with an F1 score of 0.76 at the initial stage of COVID 19. The model does not have a polyglot, so they annotated categories manually for three languages.

The study in Daley (24) used ML algorithms for the automatic classification of fake news on COVID-19. The features considered for evaluation included the count of motion words and relativity words, prepositions in the headlines of the news website, tone expressed, and word count. Also, any news media would concentrate on the increased count of these parameters to convince the customers on the fake news outlets. They attained 79% accuracy on 5-fold cross-validation using a decision tree classifier, which outperformed other algorithms in the scikit-learn API. An automated framework for detecting COVID-19 cases from X-ray images of the raw chest was proposed in Ozturk et al. (25). They used a deep neural network, namely the DarkCovidNet model (used for real-time object detection) with 17 convolution layers, each with different filters. The average age of the positive COVID patients in the dataset was 55. The model was used for binary (COVID or not) and multiclass classification (COVID or pneumonia or not) with an accuracy of 98 and 87%, respectively. The performance of the system was assessed with radiologists only, and the robustness of the system can be tested with large datasets.

Therefore, it is evident from the survey that very few attempts were carried out for detecting the fake news in COVID. Although various deep learning techniques were employed for detection, the datasets taken for analysis are very small. It is mandatory to have a strong model to detect false information on COVID-19 to avoid unwanted chaos in different aspects of COVID-19.

## 3. Proposed Architecture

In this section, the proposed work is discussed in detail.

**Figure 2:**
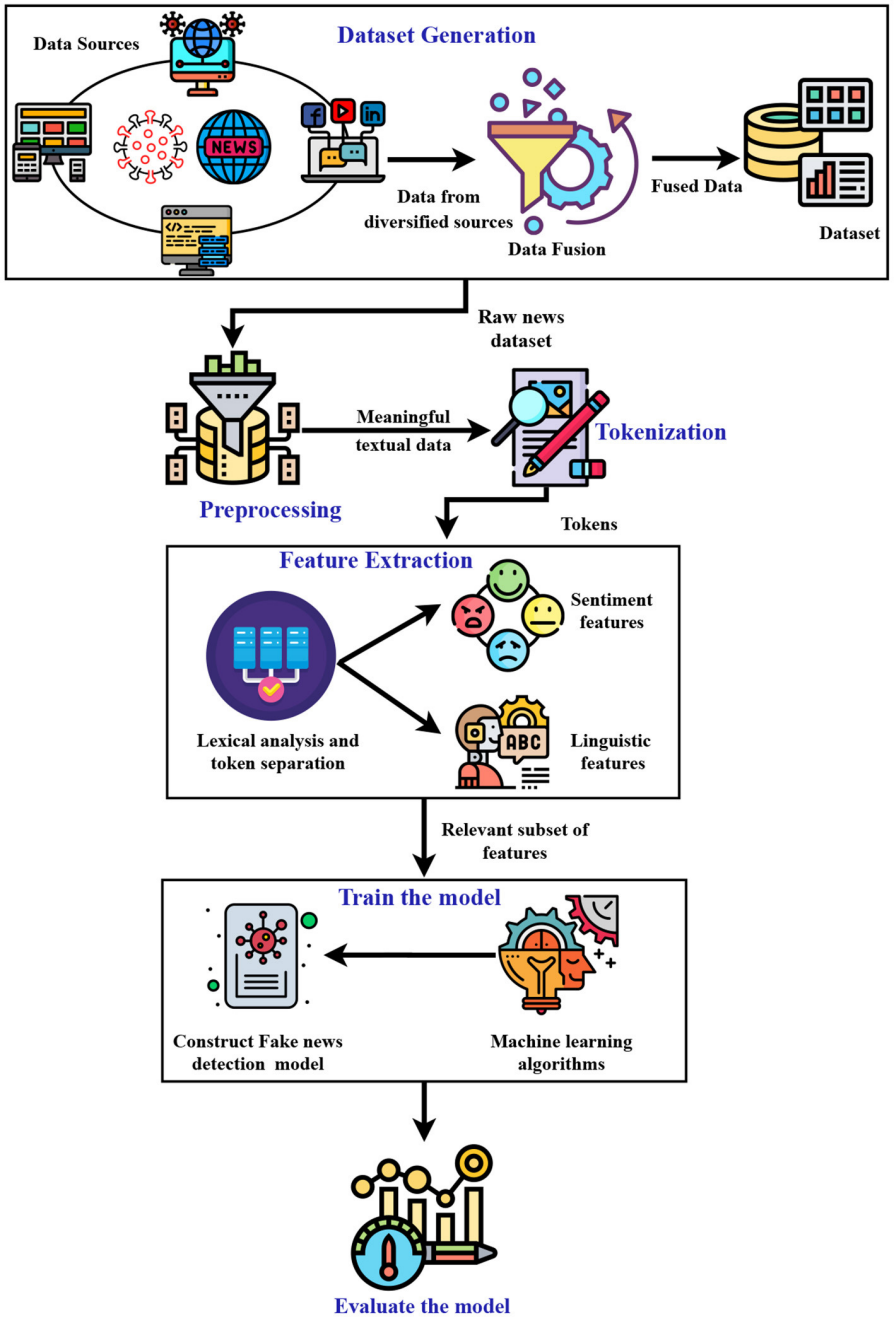
Proposed architecture.

The steps involved in the proposed work are shown in Figure 2 and are summarized as follows:

- The dataset used in this study is collected from various websites and social media cites.
- Data preprocessing is performed on the dataset by removing URLs, punctuation marks, and empty columns.
- After text preprocessing, tokenization is performed to convert the larger text into words or small lines.
- In the next step, extracting the features from text is performed.
- The features extracted are then passed on to the state-of-the-art ML learning algorithms to train the model.
- Various evaluation metrics are used to evaluate the performance of the ML algorithms.

The rest of this section discusses various steps involved in the proposed work.

### 3.1. Dataset Description

The dataset used in this work is a fusion of several fake and real news articles about COVID-19 which are collected across several platforms such as Facebook, Twitter, The New York Times, Harvard Health Publishing, WHO, etc. The dataset has 1,164 instances out of which 586 instances are true and the remaining 578 are fake news (26).

### 3.2. Preprocessing and Tokenization

The dataset considered for this work is clean. However, some unnecessary symbols which have an impact on the final classification of the news are to be removed from the dataset. To remove the unnecessary symbols, such as punctuation marks, URLs are removed from the dataset as part of preprocessing.

Tokenization is the process of splitting text into a set of tokens. The fake news dataset is tokenized to convert the long sentences into small words/tokens.

### 3.3. Feature Extraction

The major contribution of this work is the extraction of important features from the COVID-19 fake news dataset. Feature extraction plays a very important role in text processing as it reduces the dimension of feature space by considering only the important features (27–29). To extract the features, the named-entity recognition (NER) approach is used in our work. The NER is a popular approach for feature extraction that can classify unstructured text based on location, person names, quantities, etc. (30).

In this study, 39 features are created from the COVID-19-related fake news dataset. The extracted features are represented in Table 1.

**Table 1:** Features extracted from text.

**Feature name**	**Data type**	**Feature name**	**Data type**
News source	Non-Numeric	Num of?	Numeric
Num of Stopwords	Numeric	Num of /	Numeric
Num of @	Numeric	Num of #	Numeric
Num of numeric values	Numeric	Num of uppercase characters	Numeric
Num of lowercase characters	Numeric	Num of all uppercase characters	Numeric
Text language	Numeric	Word count	Numeric
Character count	Numeric	Sentence count	Numeric
Average word length	Numeric	Average sentence length	Numeric
Positive Sentiment Score	Numeric	Negative Sentiment Score	Numeric
Neutral Sentiment Score	Numeric	Compound Sentiment Score	Numeric
Person	Numeric	NORP	Numeric
FAC	Numeric	Organization	Numeric
GPE	Numeric	Location	Numeric
Product	Numeric	Event	Numeric
Work of Art	Numeric	Law	Numeric
Language	Numeric	Date	Numeric
Time	Numeric	Percent	Numeric
Money	Numeric	Quantity	Numeric
Cardinal	Numeric	Ordinal	Numeric
Text Polarity	Numeric		

### 3.4. Training of the Dataset Using ML Algorithms

In the next step, the extracted features are trained using several state-of-the-art ML algorithms. The ML algorithms used in this work are linear SVC, logistic regression, SGD classifier, random forest classifier, bagging classifier, AdaBoost classifier, decision tree classifier, and K-nearest neighbors classifier (KNN) (31). These algorithms are explained briefly in the rest of this section.

#### 3.4.1. Random Forest Algorithm

The random forest classifier is one of the supervised ML algorithms used for classification and regression purposes (32). The random forest method constructs decision trees from datasets and creates a forest made of trees. Then it collects the prediction value from each decision tree and computes the optimal solution with the help of voting. It is used as an ensemble method and helps to avoid overfitting (33). The following steps are implemented in the random forest classifier:

1. Random data samples are selected from the available dataset.
2. A decision tree is constructed for each data sample selected, and a prediction value is obtained from each decision tree. The Gini coefficient method is applied for node splitting as follows:

(1) $$\begin{array}{r} Gini\left(D\right)=1-\overset{n}{\underset{i=1}{\sum }}P_{i}^{2} \end{array}$$

where D is the sample dataset and *P*_*i*_ is the probability of decision class that can appear in D

3. A voting method is applied on the prediction values obtained from each decision tee.
4. A final prediction result is chosen from the prediction value with more votes.

#### 3.4.2. AdaBoost Classifier

The AdaBoost classifier (34) is an ensemble boosting classification method which combines several weak classifiers to build a strong classifier. The weak classifier performs the classification of one dimension of the input vector. The number of weak classifiers are increased during the training process to obtain more accuracy. The classifier applies minimum error to compute the weight of the newly added weak classifier. Then it updates the weights of each training dataset and sends the value to the recently added weak classifier. The selection of weak classifiers is very important for this algorithm. The result of the strong classifier *S*(*f*) is represented in the equation given below (35):


(2) $$\begin{array}{r} S\left(f\right)=sign\left\{\overset{T}{\underset{t-1}{\sum }}\beta_{t}h_{t}\left(f\right)\right\} \end{array}$$

where *f* denotes the input feature vector, *T* denotes the number of input vectors, *h*_*t*_(*f*) denotes weak classifiers, and β_*t*_ denotes the weights of weak classifiers.

#### 3.4.3. Decision Tree Classifier

The decision tree classifier is one of the more popular ML algorithms applied for classification and prediction problems on supervised data. It classifies the trained dataset into trees and rules. It defines methods for the classification of categorical data characterized by their factors. It works better on classification problems where extensive data are involved, and is therefore used in many data mining applications. The first step in the decision tree classifier is to find the attribute to split the tree. Entropy measure is used to identify the best attribute as the root of the decision tree. The entropy measure is used to calculate the update in homogeneity that results from the split on every attribute. Entropy D of the dataset is defined as follows:


(3) $$\begin{array}{r} Entropy\left(D\right)=\overset{n}{\underset{i=1}{\sum }}-p_{i}log_{2}\left(p_{i}\right) \end{array}$$

Where D is the data partition, n defines the number of decision classes, and *p*_*i*_ defines the proportion of the records that comes under the decision class i. Information gain is used for this calculation. This is defined as the difference between the entropy of the dataset calculated before the split (D1) and the entropy calculated from the partitioned split (D2). The formula to calculate the information gain is given as follows:


(4) $$\begin{array}{r} InformationGain\left(G\right)=Entropy\left(D_{i}\right)-Entropy\left(D_{2}\right) \end{array}$$

The attribute with maximum information gain is selected to build the tree with a root node. A decision tree is constructed recursively using the obtained data partition until there are no attributes left. And decision rules can be defined from the constructed decision tree.

#### 3.4.4. K-Nearest Neighbor Classifier

The K-nearest neighbor (KNN) (36) classifier is one of the popularly used supervised learning algorithms for classification and regression. It is a non-parametric and lazy algorithm in which the method is defined from the dataset. Also it does not require a training dataset for the development of the classification model. K is the number of nearest neighbors and it is the deciding factor. The classifier is based on the estimation of the nearest neighbor. It works on the calculation of a similarity value defined by the distance measure. The training step stores all the instances and their corresponding class labels. The test phase for a new instance 's' is calculated using the following steps:

1. Calculate the distance between the instance 's' in the test data and every instance in the training dataset 'd' using the Euclidean distance formula given as follows:

(5) $$\sqrt{\sum_{i=1}^{n}{\left(x_{i}-y_{i}\right)}^{2})}$$

where n is the total number of instances in the dataset, *x*_*i*_ represents instances in the training dataset, and *y*_*i*_ are instances in the test dataset 'd'.

2. Arrange the distances in ascending order and select the first 'k' instances.
3. Find the most frequent decision class in the calculated 'k' nearest neighbors.

## 4. Experiment Results

The experiments on the COVID-19 fake news dataset are carried out in Google Colab, an open source cloud-based graphical processing unit (GPU)-based platform offered by Google Inc. The programming language used to implement the ML algorithms is Python 3.7.

A total of 70% of the COVID-19 fake news dataset is used for training the ML algorithms and the other 30% of the dataset is used for validating and testing the ML algorithms. The performance of the ML algorithms is evaluated on several metrics like accuracy, precision, recall, F1-measure, prediction time, and training time.

The rest of this section discusses the results obtained by the ML algorithms on the COVID-19 fake news dataset before and after the feature extraction.

### 4.1. COVID-19 Fake News Experiment Results Before Feature Extraction

**Figure 3:**
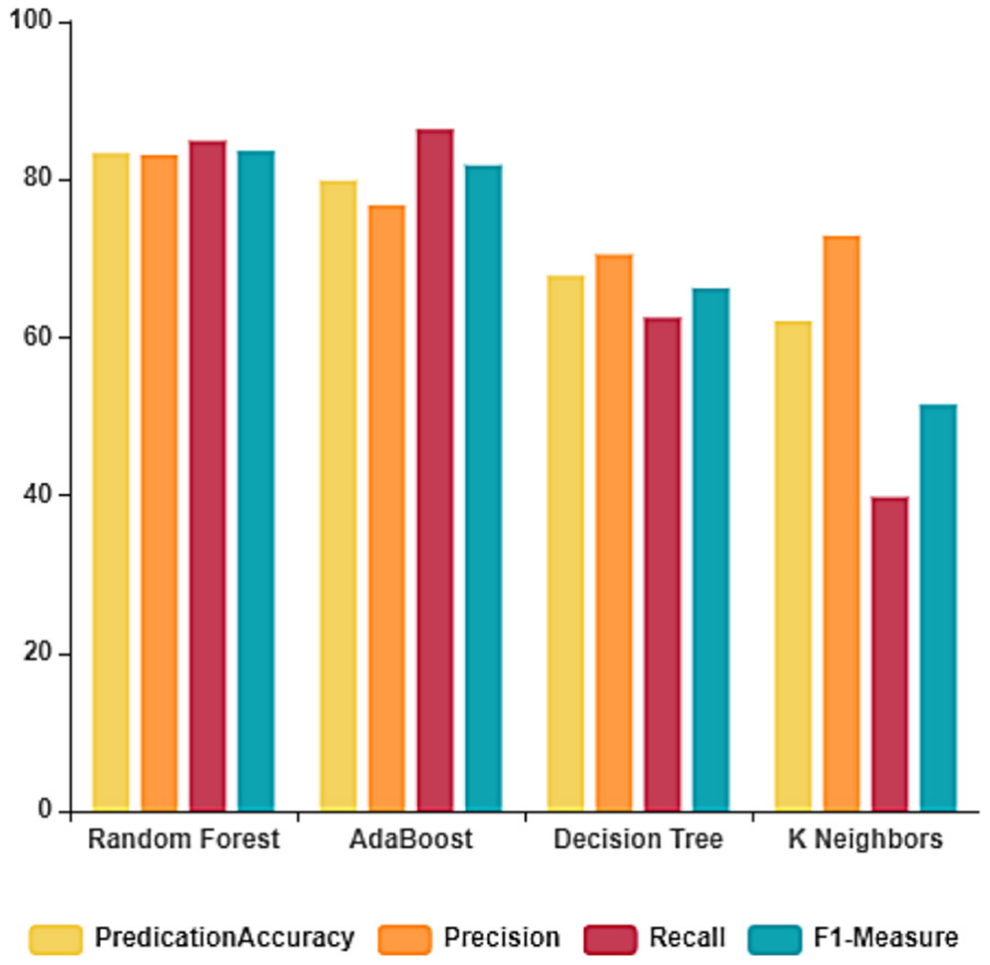
Classification report before feature extraction.

**Figure 4:**
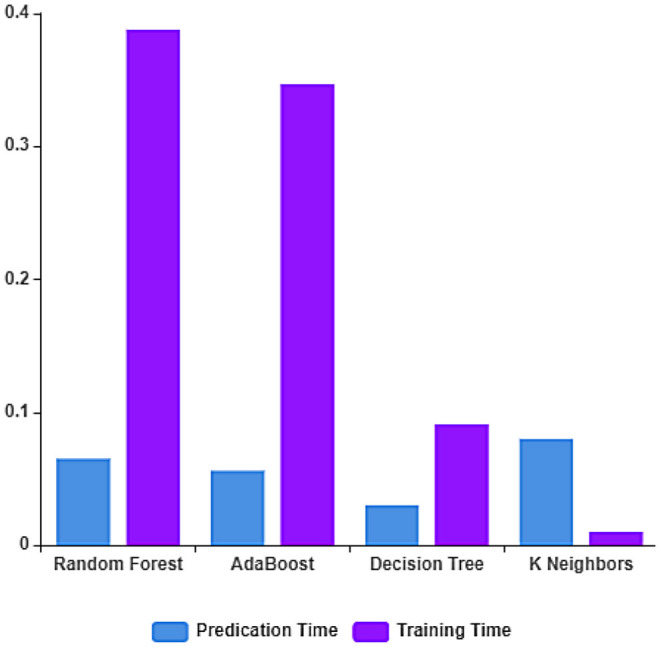
Training and testing time rate before feature extraction.

The performance of the ML algorithms on the COVID-19 fake news dataset before the feature extraction is depicted in Figure 3 and Table 2. From the table, it is evident that the random forest classifier achieves better prediction accuracy, precision, recall, and F1-measure with 83.33, 83.14, 84.90, and 83.61%, respectively. The random forest classifier trains the dataset in the least time when compared to other ML algorithms. The training and testing time of the ML algorithms on the dataset considered before feature extraction is depicted in Figure 4.

**Table 2:** Performance of the ML algorithms before feature extraction.

**Algorithm**	**Predication accuracy**	**Precision**	**Recall**	**F1-measure**	**Predication time (S)**	**Training time (S)**
Random forest classifier	83.33	83.14	84.90	83.61	0.065862	0.383069
AdaBoost classifier	79.88	76.76	86.36	81.82	0.056415	0.347593
Decision tree classifier	67.81	70.51	62.50	66.26	0.003247	0.091995
K-nearest neighbor classifier	62.06	72.91	39.77	51.47	0.084975	0.001555

### 4.2. COVID-19 Fake News Experiment Results After Feature Extraction

**Figure 5:**
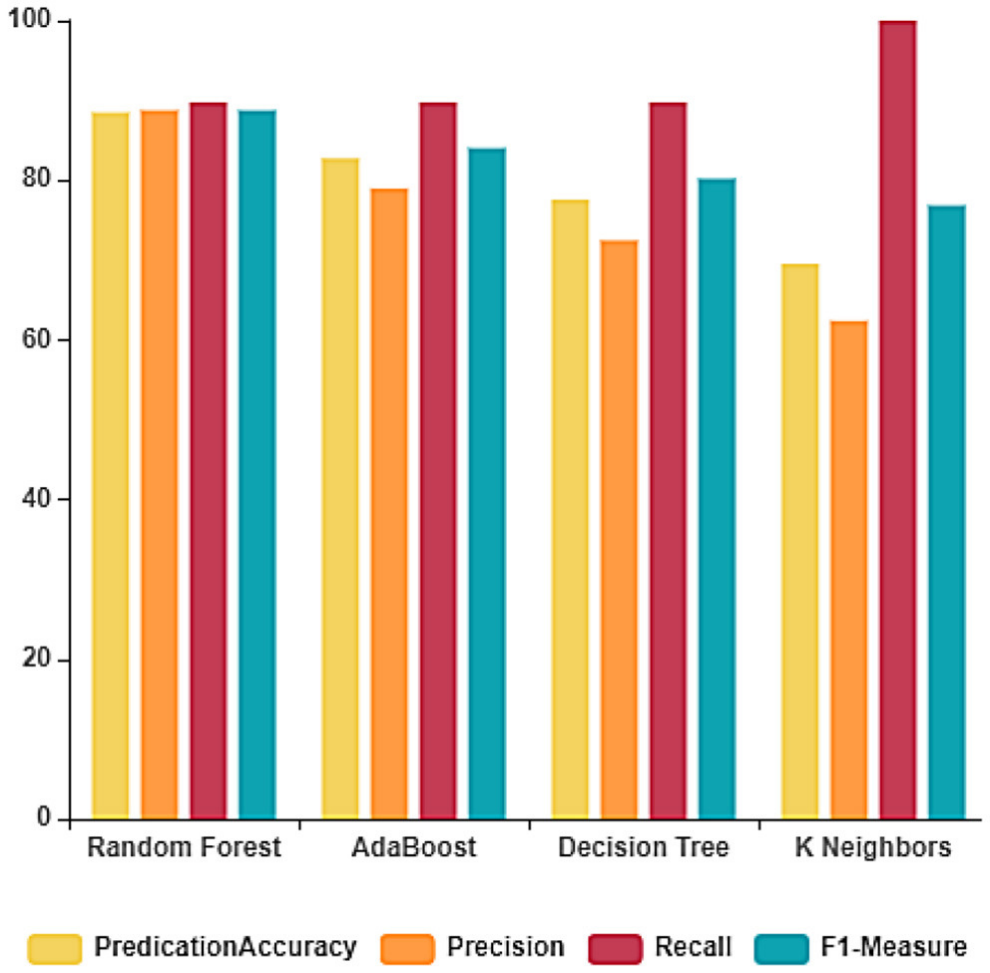
Classification report after feature extraction.

The performance of the ML algorithms on the COVID-19 fake news dataset after the feature extraction is depicted in Figure 5 and Table 3. From these results, it is evident that the random forest classifier outperforms the other classifiers in terms of accuracy and precision with 88.50 and 87.77%, respectively. Whereas, linear SVC achieved better recall and F1-measure with 89.77 and 88.76%, respectively.

**Table 3:** Performance of the ML algorithms after feature extraction.

**Algorithm**	**Predication accuracy**	**Precision**	**Recall**	**F1-measure**	**Predication time (S)**	**Training time (S)**
Random forest classifier	88.50	87.77	89.77	88.76	0.004685	0.011387
AdaBoost classifier	82.75	79.00	89.77	84.04	0.004750	0.053603
Decision tree classifier	77.58	72.47	89.77	80.20	0.002851	0.081209
K-nearest neighbor classifier	69.54	62.41	100	76.85	0.003293	0.008997

**Figure 6:**
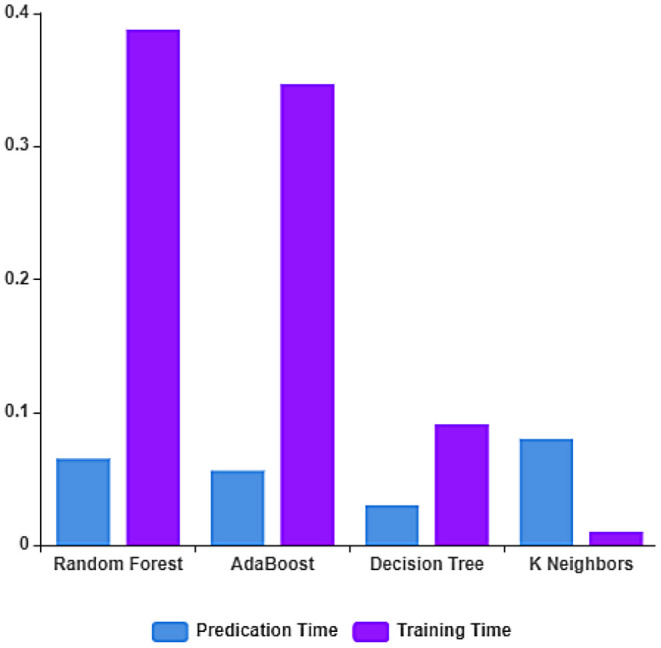
Training and Testing time rate after feature extraction.

The training and prediction time of the ML algorithms on the dataset considered after feature extraction is depicted in Figure 6. From the figure, it can be observed that the decision tree classifier has the least prediction time and the linear SVC classifier has the least training time.

## 5. Discussion

From the results section, it is clear that the performance of the machine learning algorithms improves after they are trained with extracted features from the COVID-19 fake news dataset. Since the size of the dataset is approximately 1,100 records, ML algorithms are chosen for classification rather than deep neural network-based algorithms.

The raw fake news dataset has several words that have no affect on the classification results. When the ML algorithms are trained by the raw dataset without feature extraction, there is a very high chance that the performance of the ML algorithms will be affected by some of the frequent words in the text that have no effect on the classification results. Hence, in this work, we employed NER to extract features from the fake news dataset. The extracted features are then fed to the ML algorithms for training. The comparison between the results of the ML algorithms before and after the feature extraction prove that the performance of the ML algorithms increase after feature extraction.

## 6. Conclusion

In this work, a sophisticated model that extracts important features from a COVID-19 fake news dataset is presented that improves the performance of ML algorithms. Social media applications are the most significant source to spread any kind of information. So in his work, the dataset related to COVID-19 which was the fusion of several diversified social media platforms and news websites is used for evaluating the performance of the proposed model. A data preprocessing step is applied on the dataset to remove several unnecessary symbols like tags and URLs from the dataset. Later on, to enhance the performance of the machine learning algorithms, the relevant subset of features categorized into linguistic and sentiment features are extracted using the NER approach. The extracted features are trained using several state-of-the-art ML algorithms, namely the random forest classifier, AdaBoost classifier, decision tree classifier, and KNN classifier. The performance of the ML algorithms is analyzed before and after the feature extraction. Random forest provides better accuracy at 83.33% without feature extraction, whereas KNN trains the dataset faster than others. Of several ML algorithms, the random forest classifier with feature extraction outperforms the others with an accuracy of 88.50%, precision of 87.77%, recall of 89.77%, and F1 score of 88.76%. The experiment results prove the importance of feature extraction in fake news detection.

Further in future, audio clips, images, and video clips can be considered for generating fake news data as they are the most attractive way to spread fake information. Deep learning algorithms and natural language processing for the semantical understanding of linguistic features can be further analyzed on a dataset containing text, images, audio, and video.

## Data Availability Statement

The datasets presented in this study can be found in online repositories. The names of the repository/repositories and accession number(s) can be found in the article/Supplementary Material.

## Author Contributions

All authors listed have made a substantial, direct, and intellectual contribution to the work and approved it for publication.

## Conflict of Interest

The authors declare that the research was conducted in the absence of any commercial or financial relationships that could be construed as a potential conflict of interest.

## Publisher's Note

All claims expressed in this article are solely those of the authors and do not necessarily represent those of their affiliated organizations, or those of the publisher, the editors and the reviewers. Any product that may be evaluated in this article, or claim that may be made by its manufacturer, is not guaranteed or endorsed by the publisher.
