# Supplementary figures and images for: Detecting COVID-19-Related Fake News Using Feature Extraction
Source: Front Public Health. 2022 Jan 4;9:788074. doi: 10.3389/fpubh.2021.788074 (PMC8764372; doi:10.3389/fpubh.2021.788074)

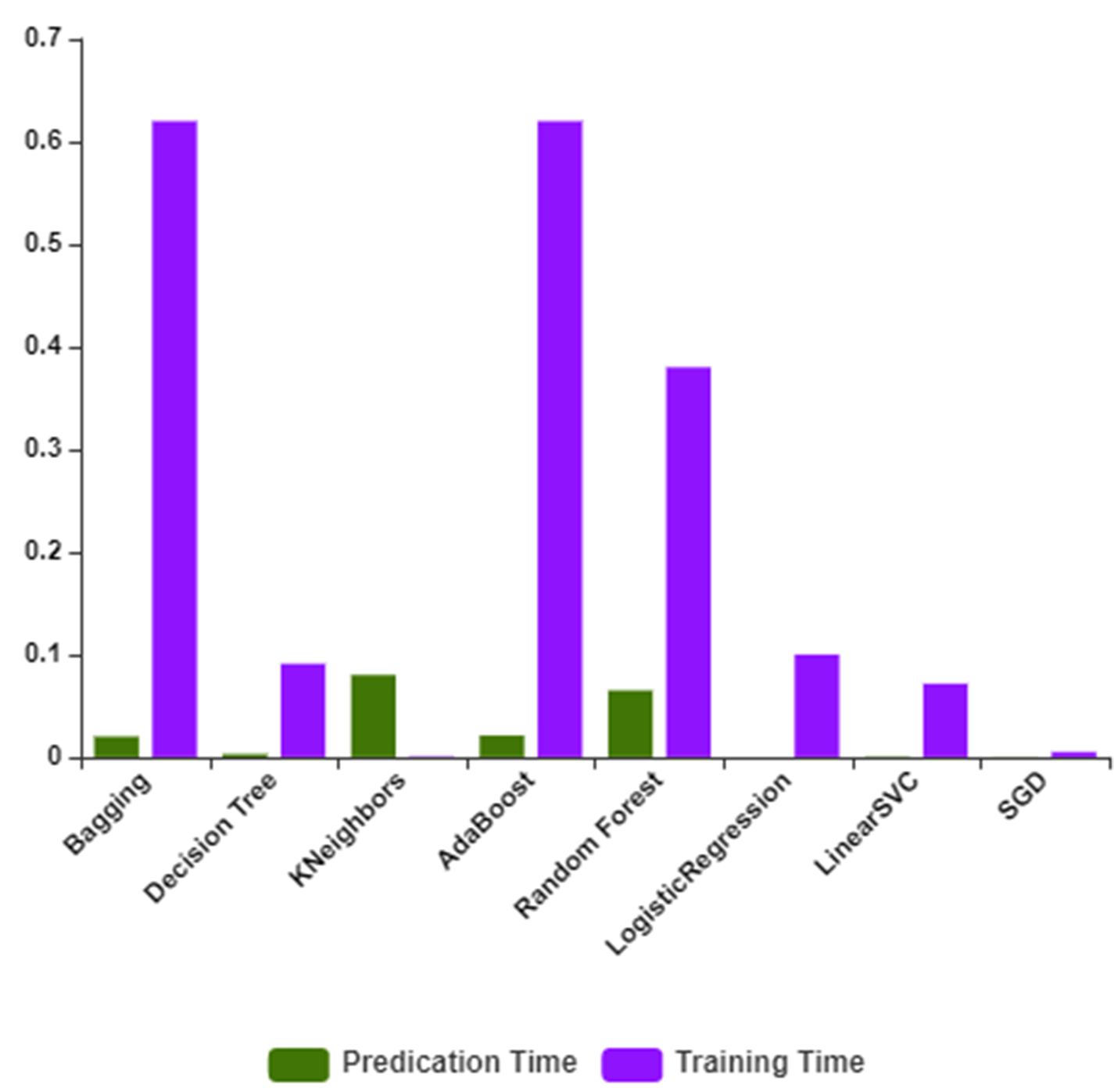

Supplement: Supplementary file 1 [file Image_1.PNG]

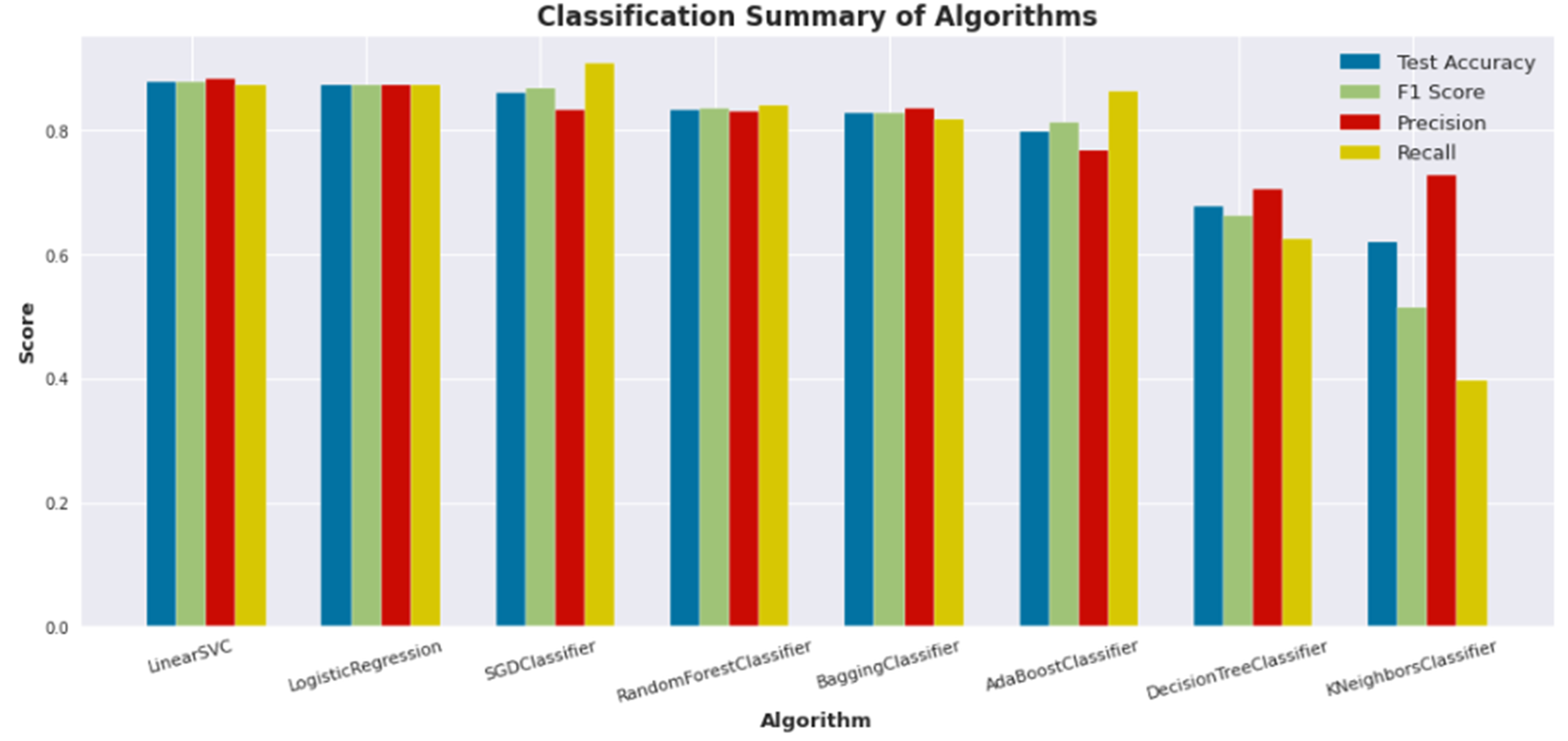

Supplement: Supplementary file 2 [file Image_4.PNG]

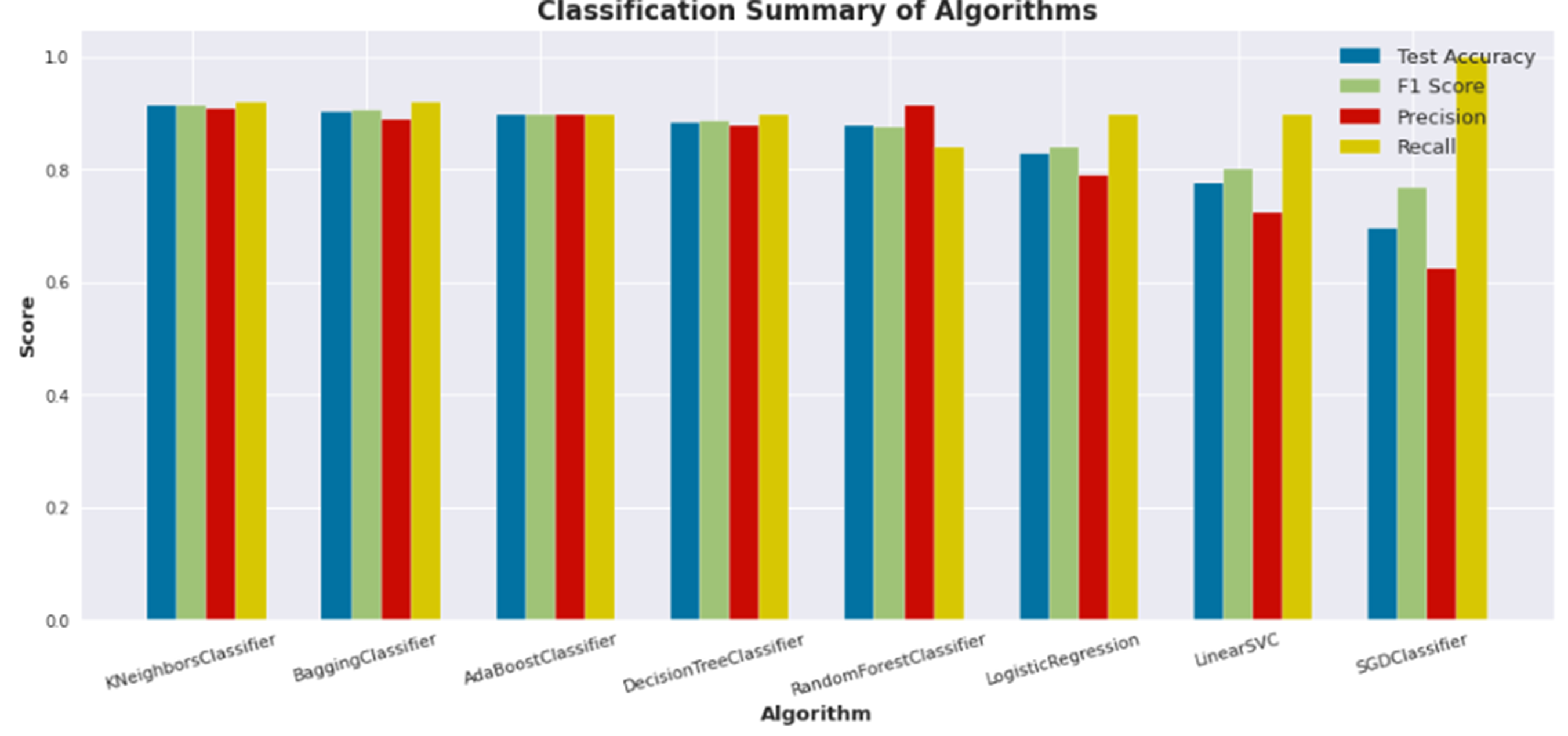

Supplement: Supplementary file 3 [file Image_5.PNG]

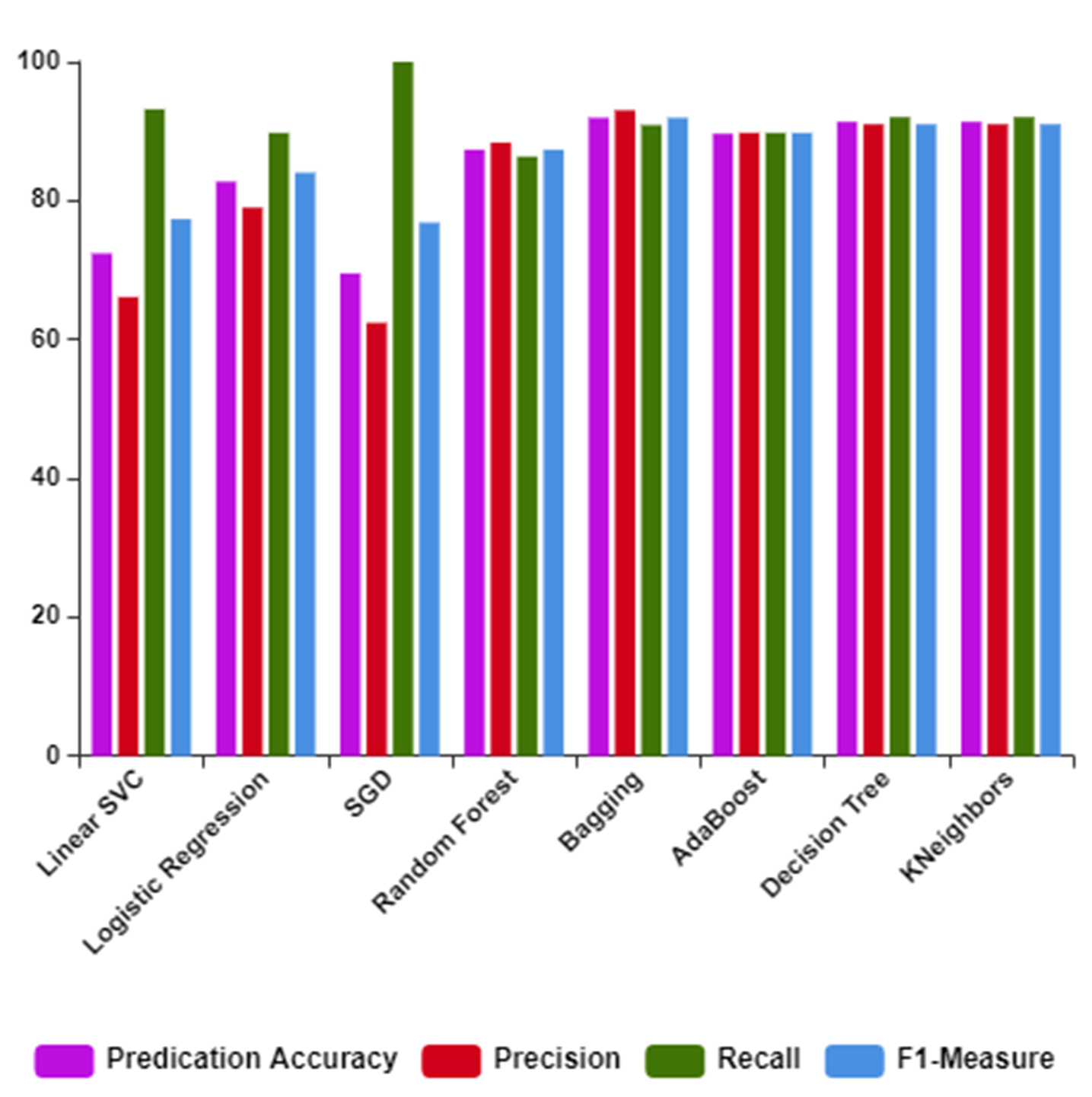

Supplement: Supplementary file 4 [file Image_8.PNG]

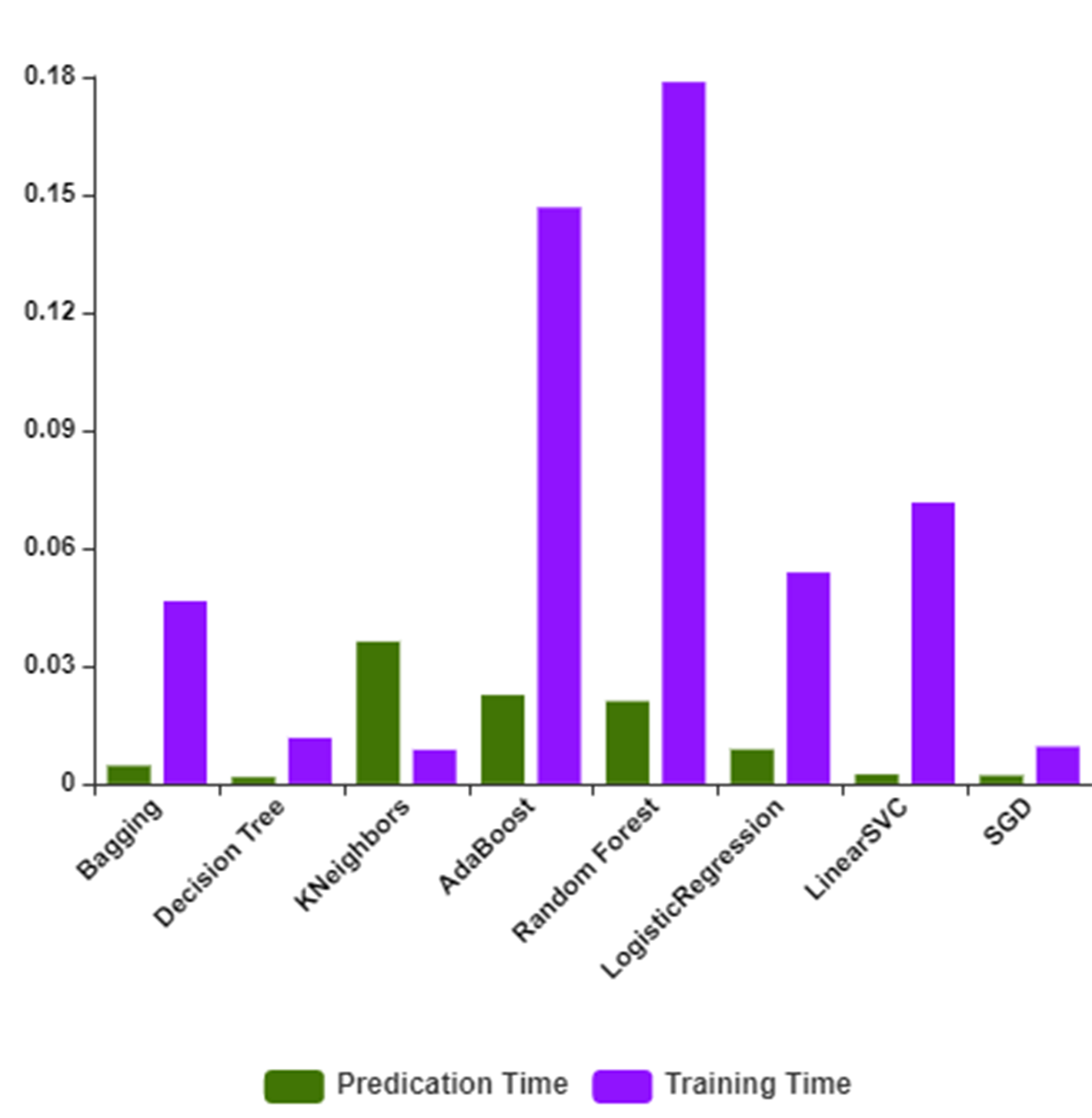

Supplement: Supplementary file 5 [file Image_9.PNG]

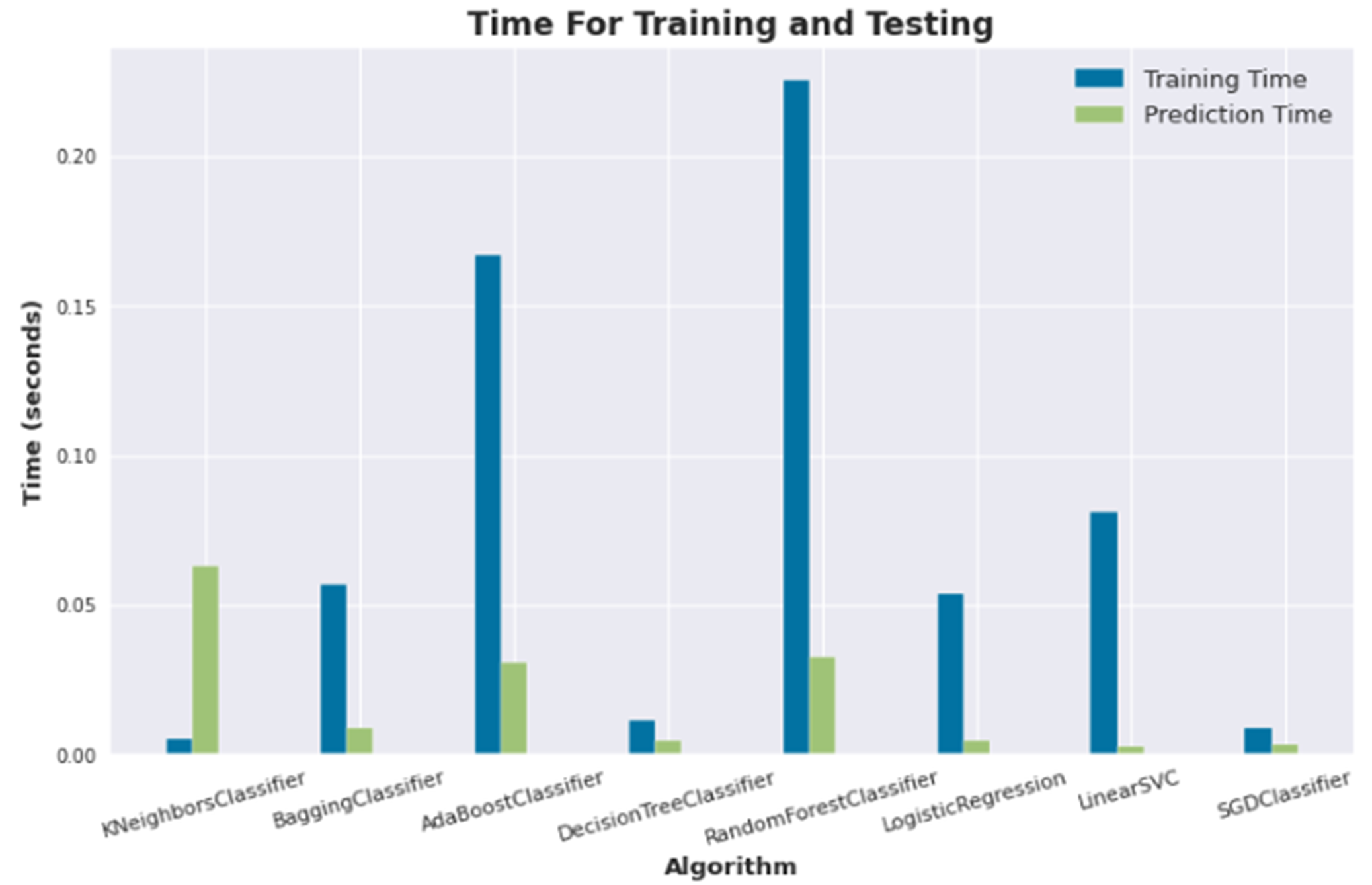

Supplement: Supplementary file 6 [file Image_10.PNG]

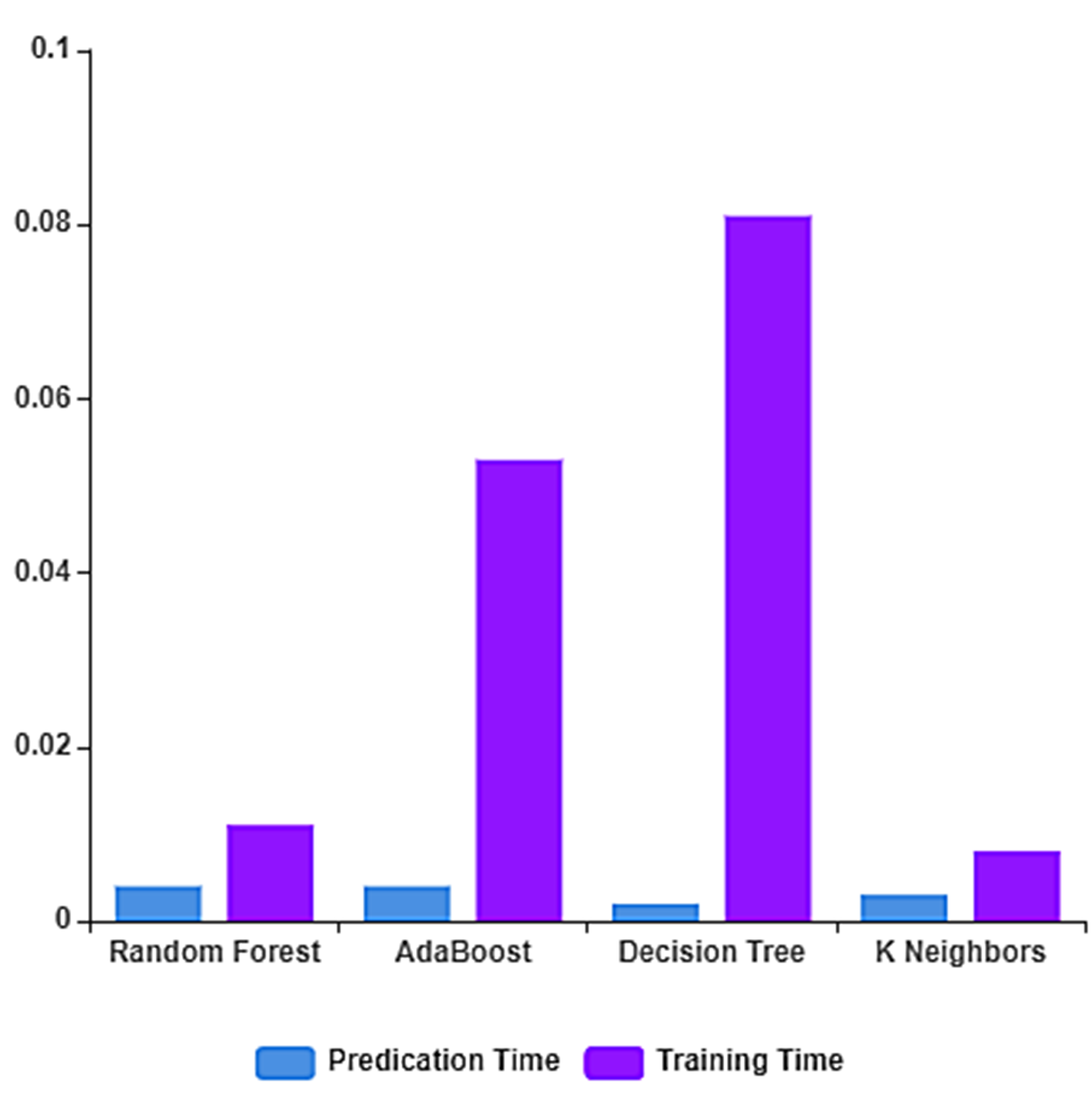

Supplement: Supplementary file 7 [file Image_12.PNG]

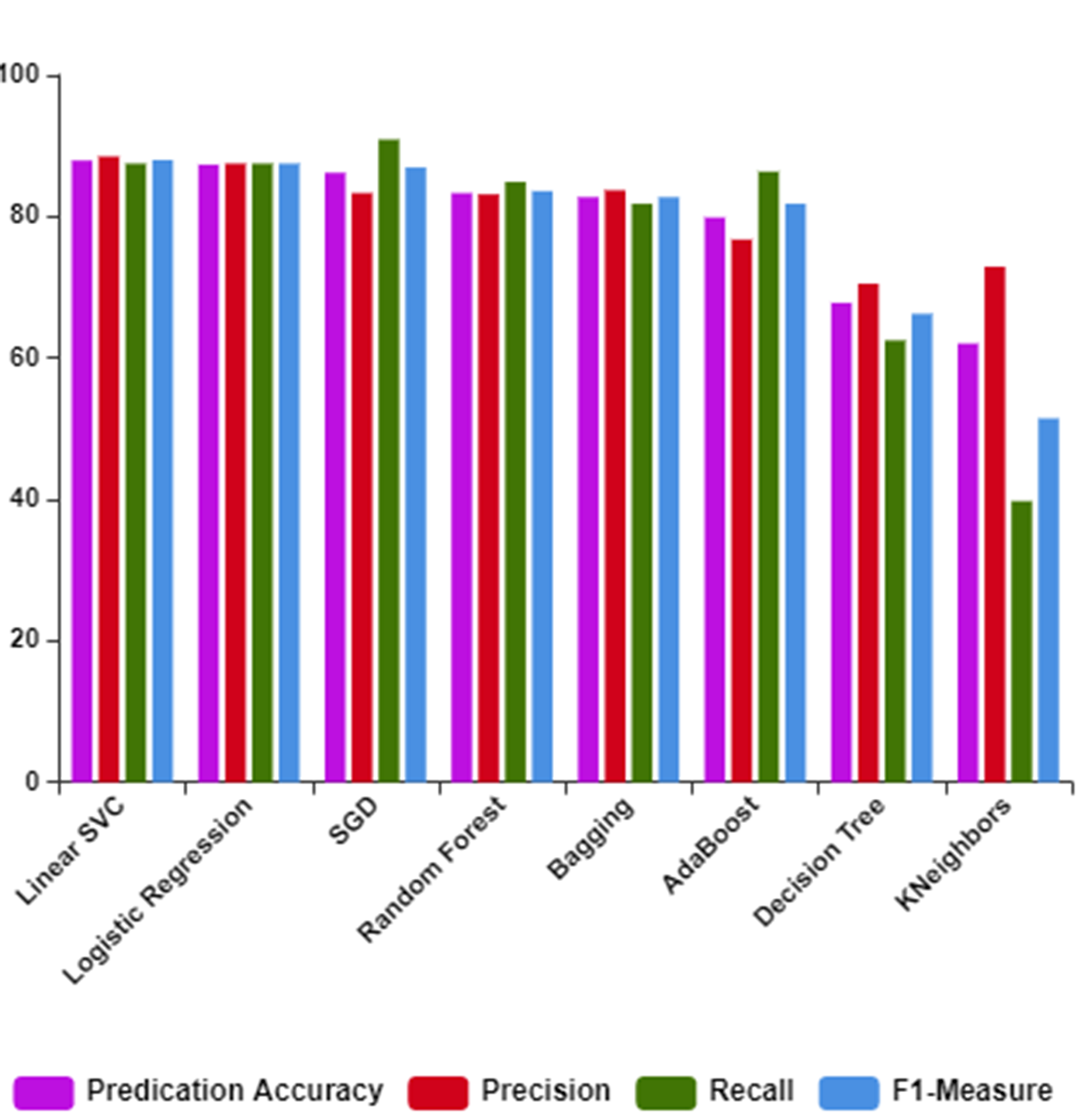

Supplement: Supplementary file 8 [file Image_13.PNG]
